# Enhancement of doxorubicin anti-cancer activity by vascular targeting using IsoDGR/cytokine-coated nanogold

**DOI:** 10.1186/s12951-021-00871-y

**Published:** 2021-05-05

**Authors:** Angelo Corti, Angelina Sacchi, Anna Maria Gasparri, Matteo Monieri, Giulia Anderluzzi, Barbara Colombo, Alessandro Gori, Anna Mondino, Flavio Curnis

**Affiliations:** 1 Tumor Biology and Vascular Targeting Unit, Division of Experimental Oncology, IRCCS San Raffaele Scientific Institute, via Olgettina 58, 20132 Milan, Italy; 2 Università Vita-Salute San Raffaele, Milan, Italy; 3 Istituto di Scienze e Tecnologie Chimiche, C.N.R., Via Mario Bianco 9, 20131 Milan, Italy; 4 Lymphocyte Activation Unit, Division of Immunology, Transplantation and Infectious Diseases IRCCS San Raffaele Scientific Institute, Milan, Italy

**Keywords:** *Iso*Asp-Gly-Arg (*iso*DGR motif), Αvβ3 integrin, TNF, IL12, Gold nanoparticles, Tumor vascular targeting

## Abstract

**Background:**

Gold nanospheres tagged with peptides containing *iso*DGR (*iso*Asp-Gly-Arg), an αvβ3 integrin binding motif, represent efficient carriers for delivering pro-inflammatory cytokines to the tumor vasculature. We prepared bi- or trifunctional nanoparticles bearing tumor necrosis factor-α (TNF) and/or interleukin-12 (IL12) plus a peptide containing isoDGR, and we tested their anti-cancer effects, alone or in combination with doxorubicin, in tumor-bearing mice.

**Results:**

In vitro biochemical studies showed that both nanodrugs were monodispersed and functional in terms of binding to TNF and IL12 receptors and to αvβ3. In vivo studies performed in a murine model of fibrosarcoma showed that low doses of bifunctional nanoparticles bearing isoDGR and TNF (corresponding to few nanoparticles per cell) delayed tumor growth and increased the efficacy of doxorubicin without worsening its toxicity. Similar effects were obtained using trifunctional nanoparticles loaded with isoDGR, TNF and IL12. Mechanistic studies showed that nanoparticles bearing isoDGR and TNF could increase doxorubicin penetration in tumors a few hours after injection and caused vascular damage at later time points.

**Conclusion:**

*Iso*DGR-coated gold nanospheres can be exploited as a versatile platform for single- or multi-cytokine delivery to cells of the tumor vasculature. Extremely low doses of *iso*DGR-coated nanodrugs functionalized with TNF or TNF plus IL12 can enhance doxorubicin anti-tumor activity.

**Graphic Abstract:**

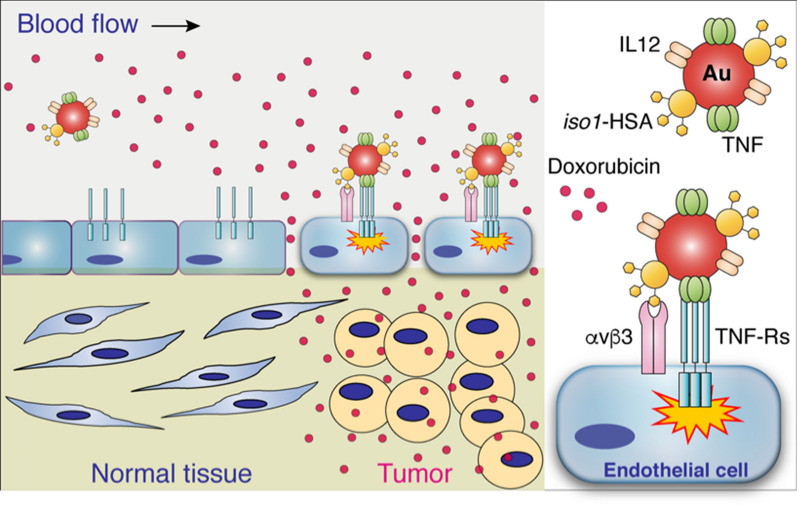

**Supplementary Information:**

The online version contains supplementary material available at 10.1186/s12951-021-00871-y.

## Background

The major limitation in using cytokines for cancer therapy is often related to their prohibitive toxicity and rapid induction of counter-regulatory mechanisms [1–3]. A growing body of evidence suggests that these obstacles can be overcome by strategies based on ligand-directed delivery of low-dose cytokines to tumors [4–7]. This approach can be achieved by cytokine conjugation to antibodies or peptide ligands that recognize specific receptors expressed by tumor cells or by other elements of the tumor microenvironment, including tumor vessels [4, 6]. For example, peptides containing Ans-Gly-Arg (NGR), Arg-Gly-Asp (RGD), or *iso*Asp-Gly-Arg (*iso*DGR) sequences selective for CD13 or integrins overexpressed by the tumor vasculature have been exploited as ligands for delivering tumor necrosis factor-α (TNF) [7], interferon (IFN) α2a [8], or IFNγ [9] to tumors, consequently improving their therapeutic index [7, 10]. A prototypical compound belonging to this class of molecules is NGR-TNF, a peptide-cytokine conjugate that alters endothelial permeability and enhances the penetration of chemotherapeutic drugs in tumor tissues [3, 7]. Low doses of this drug have been tested in various clinical studies in patients with solid tumors, including patients with primary lymphomas of the central nervous system (PCNSL), with evidence of activity and good tolerability [7, 11–14]. Notably, in these patients, NGR-TNF altered the blood–brain barrier and increased the efficacy of R-CHOP, a chemo-immunotherapeutic regimen [13, 14]. The therapeutic index of TNF and other cytokines can also be increased by coupling cytokines with colloidal gold (Au), a well-tolerated nanomaterial that accumulates in tumors by “passive” targeting mechanisms consequent to the enhanced permeability and retention (EPR) effects of abnormal vessels of tumor tissues [15–19]. We have recently shown that these “active” and “passive” targeting approaches (mediated by peptides and nanogold, respectively) can be combined to generate more efficient cytokine delivery systems. For example, we have shown that NGR-, methylated NGR-, or *iso*DGR-tagged gold nanoparticles represent efficient carriers for delivering TNF or IL12 to tumors [20–23]. Indeed, in animal models of solid tumors, these nanocarriers can deliver pharmacologically active amounts of TNF or IL12 to tumors, with no evidence of toxicity [20–23]. However, whether these targeted nanodrugs synergize with chemotherapy remains to be determined. The present study was undertaken to explore the capability of *iso*DGR-coated nanogold bearing TNF and/or IL12, alone or in combination, to synergize with chemotherapy. The rationale for exploring the TNF/IL12 combination, in addition to TNF alone, relies on the notion that IL12 can induce IFNγ in the tumor microenvironment [24, 25], a cytokine known to be critical for the activity of vasculature-targeted TNF [26]. Thus, we prepared gold nanoparticles (Nps) bearing TNF and/or IL12 and c(CG*iso*DGRG) (*iso1*), a head-to-tail cyclic peptide that recognizes αvβ3 integrin overexpressed in the tumor vasculature [27, 28]. We also tested their physicochemical, biochemical and biological properties in vitro and their therapeutic activity in a murine model of fibrosarcoma, alone or combined with doxorubicin (a chemotherapeutic drug). We show that an extremely low dose of nanogold functionalized with *iso1* and TNF (bifunctional) or with *iso1*, TNF and IL12 (trifunctional) can increase the efficacy of doxorubicin without increasing its toxicity. Furthermore, we show that nanogold functionalized with *iso1* and TNF increases doxorubicin penetration in tumors a few hours after injection and causes vascular damage at later time points.

## Methods

### Reagents

The following reagents were purchased: human serum albumin (HSA) 20% solution (Baxter, Deerfield, IL); bovine serum albumin (BSA) (Sigma); human integrin αvβ3 (Immunological Sciences, Italy); methoxy-PEG-SH (MW 20 KDa, PEG) (Nanocs Inc, USA); soluble TNF receptor Type-II (sTNF-R2) fused to the Fc fragment of an IgG1 antibody (Enbrel) (Pfizer, Italy); monoclonal antibody (mAb) anti-IL12/IL23, p40 subunit, clone C15.6 and C17.8 (Biolegend, USA); recombinant murine interleukin-12 (IL12) (PeproTech); and 25 nm gold (Au) nanospheres (nanogold Aurion). Recombinant murine TNF and synthetic cyclic head-to-tail c(CG*iso*DGRG) peptide (called *iso1*) were prepared as described previously [20, 29]. *Iso1*-HSA conjugate, comprising *iso1* chemically coupled to HSA via 4-(*N*-maleimidomethyl)cyclohexane-1-carboxylic acid 3-sulfo-*N*-hydroxysuccinimide ester sodium salt (sulfo-SMCC) and SMCC-HSA (HSA activated with sulfo-SMCC and quenched with β-mercaptoethanol in place of *iso1*), were prepared as described previously [20].

### Preparation of bifunctional gold nanoparticles bearing *iso1* and IL12 or *iso1* and TNF

Bifunctional gold Nps loaded with *iso1* and IL12 (called *iso1*Au/IL12) or *iso1* and TNF (*iso1*Au/TNF) were prepared, as described previously, by incubating 1 ml of 25-nm nanogold (A_520nm_, ~ 1 unit) with 0.1 ml of solution containing 120 µg of *iso1*-HSA and 2.7 µg of IL12 or 160 µg of *iso1*-HSA and 16 µg of TNF, respectively [21, 23] (see also Additional file 1: Scheme S1). Monofunctional Nps lacking *iso1* (Au/TNF and Au/IL12) were prepared in the same manner, except that SMCC-HSA was used instead of *iso1*-HSA. Other monofunctional Nps lacking cytokines (*iso1*Au) were prepared in the same way by omitting the cytokine in the incubation mixture.

### Preparation of trifunctional gold nanoparticles bearing *iso1*, IL12 and TNF

Trifunctional gold Nps loaded with *iso1*, IL-12, and TNF (called *iso1*Au/TNF + IL12) were prepared as follows: a solution containing 132 µg of *iso1*-HSA, 1 µg of TNF (19 pmol), 6 µg of IL12 (86 pmol, corresponding to TNF:IL12 molar ratio of 1:4.5), and 5 µg of PEG in 100 µl of 5 mM sodium citrate buffer, pH 6.0, was added to 1 ml of 25-nm nanogold (Aurion), with the pH adjusted to 4.3–4.4 with orthophosphoric acid (final pH ~ 5.5), and left to incubate for 2 h at room temperature under shaking. The product was mixed with 100 µl of 10% HSA in water (added in aliquots every 2 min) and incubated for an additional 5 min to saturate gold Nps. The final product was centrifuged (14,000×*g* for 15 min at 4 °C), resuspended in 1 ml of 1% HSA in 5 mM sodium citrate buffer, pH 6.0 (three times), filtered (0.22 µm pore size; Millex-GV Filter) and stored at − 80 °C (see also Additional file 1: Scheme S1). A similar product was prepared using a TNF:IL12 molar ratio of 4:1. Furthermore, Nps lacking *iso1* (called Au/TNF + IL12) were prepared in the same manner, except that SMCC-HSA was used instead of *iso1*-HSA.

### Physicochemical characterization of nanodrugs

Visible spectra of the nanodrugs were recorded using an UltroSpec 2100 spectrophotometer (Amersham Biosciences) and a 1-cm-path-length quartz cuvette. The concentration of Nps was calculated by interpolating the A_530_ nm values on a calibration curve obtained using uncoated nanogold in 5 mM sodium citrate buffer, pH 6.0 (stock solution: 3.3 × 10^11^ Nps/ml; A_530_ nm: 0.96 U/ml).

Dynamic light scattering (DLS) measurements were performed using a DLS DYNAPRO 99 instrument (Wyatt) operating with the laser intensity set to 20% power. The nanodrugs were diluted 1:10 in 5 mM sodium citrate buffer, pH 6.0 (1–3 × 10^10^ Nps/ml) and analyzed using 25–30 independent measurements of 10-s duration at 20 °C. The calculation of the hydrodynamic radius of the Nps was performed using DLS regularization analysis.

Transmission electron microscopy (TEM) analysis were performed using a TALOS L120C microscope (ThermoScientific) as described previously [23]. Morphometric analysis of nanoparticles shape and diameter was performed using the ImageJ software, essentially as previously reported [30].

### αvβ3 integrin, anti-IL12 antibody and sTNF-R2 binding assays

The binding assay of nanodrugs to αvβ3, anti-IL12 antibodies or sTNF-R2 (Enbrel) spotted onto nitrocellulose filters (0.45 µm) was performed using a Bio-Dot apparatus (Bio-Rad) as described previously [20, 21, 23]. The αvβ3/anti-IL12 antibody and αvβ3/anti-TNF antibody sandwich assays were performed as described previously [21, 23]. Briefly, various amounts of nanodrugs diluted in binding buffer (25 mM Tris–HCl, pH 7.4, containing 150 mM sodium chloride, 1 mM magnesium chloride, 1 mM manganese chloride 1% w/v BSA) were added to microtiter plates coated with or without αvβ3 (0.5 µg/ml). The binding of nanodrugs was then detected using a biotinylated anti-IL12 antibody (anti-p40 subunit; mAb C17.8) or an anti-TNF polyclonal antibody, followed by streptavidin-peroxidase or goat anti-rat antibody-peroxidase conjugates, respectively. Bound peroxidase was detected by adding the o-phenylenediamine chromogenic substrate.

### IL12- and PEG-ELISA

The amount of IL12 and PEG bound to gold Nps was quantified using the MAX Deluxe Set Mouse IL12 (p70) ELISA kit (Biolegend) according to the manufacturer’s instructions and the Polyethylene Glycol Backbone ELISA Kit (Life Diagnostics, Inc.) as described previously [23].

### In vitro IL12 and TNF bioassay

The amount of bioactive IL12 bound to gold Nps was determined using an in vitro bioassay based on IL12-induced release of IFNγ from murine splenocytes [23]. IFNγ was detected using the mouse IFN-γ DuoSet ELISA kit (R&D System). The amount of bioactive TNF bound to gold Nps was determined using an in vitro bioassay based on TNF-induced cytolysis of murine L–M fibroblasts, as described previously [29].

### In vivo studies

Murine WEHI-164 fibrosarcoma cells (CRL-1751, ATCC) were cultured in DMEM with standard supplements and tested for mycoplasma contamination before their use in vivo using the MycoAlert assay Control Set (Lonza). BALB/c mice (Charles River Laboratories; 6–8 weeks old), weighing 18 to 20 g, were challenged by s.c. injection into the left flank with 10^6^ WEHI-164 cells. Five days later, the mice were injected (i.v.) with nanodrugs in 0.9% sodium chloride containing 100 µg/ml of HSA, alone or in combination with 150 µg of doxorubicin (Adriamycin; Pfizer) in 0.9% sodium chloride. Tumor growth was monitored by measuring the tumor size with a caliper. The tumor volumes were estimated by calculating r1 × r2 × r3 × 4/3π, where r1 and r2 are the longitudinal and lateral radii, respectively, and r3 is the thickness of the tumor protruding from the surface of normal skin. Animals were sacrificed before the tumors reached a diameter of 1–1.5 cm. The tumor sizes are shown as the mean ± SE.

## Results

### Gold nanoparticles are loaded with *iso1*, TNF and/or IL12 to generate bi- or trifunctional nanodrugs

**Fig. 1:**
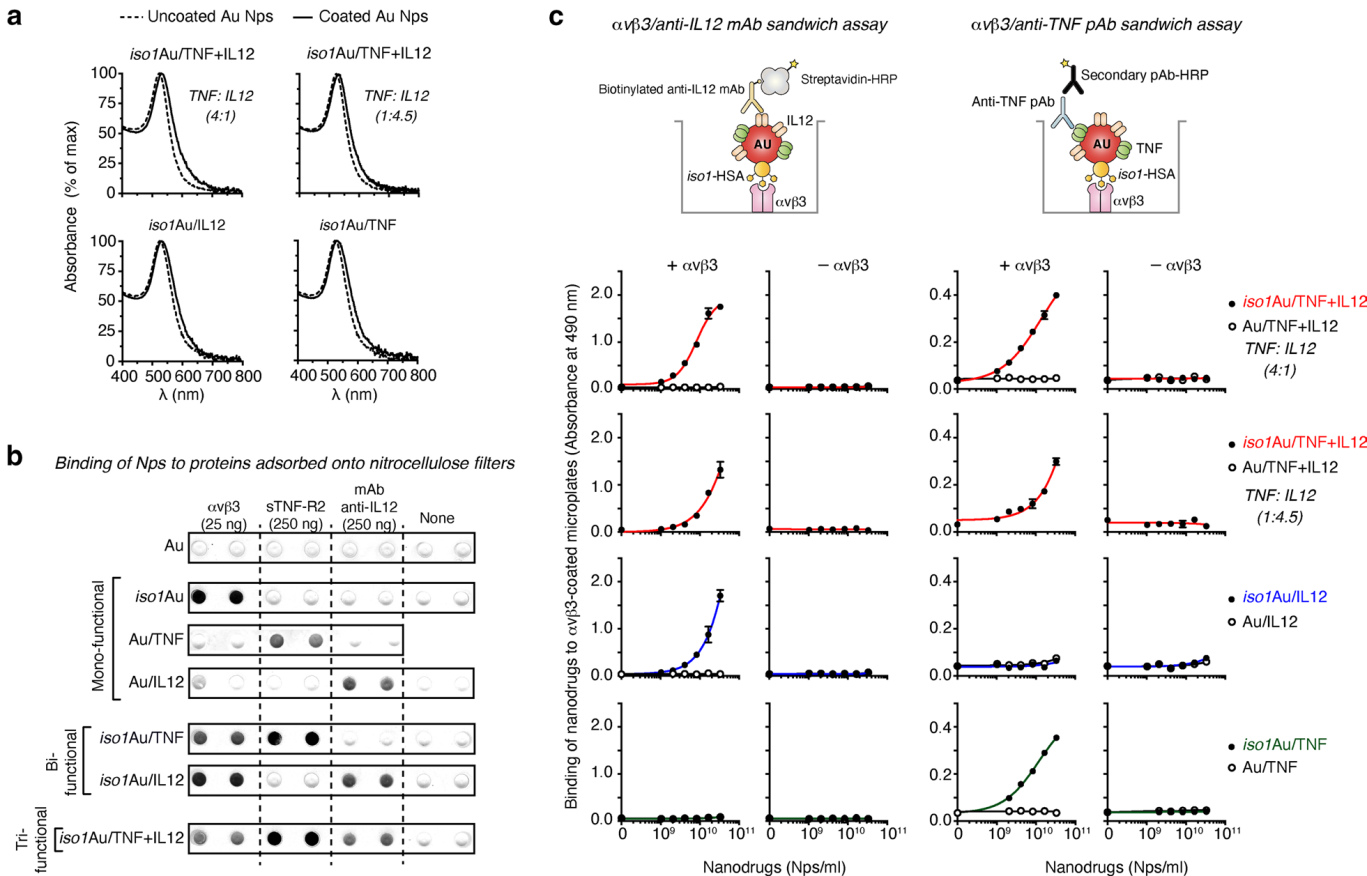
Characterization of tri- and bifunctional nanodrugs by UV–Vis spectrophotometry and binding assays to αvβ3, sTNF-R2 and anti-IL12 antibodies. **a** UV–Vis absorption spectra of nanodrugs. The dotted line and solid line correspond to uncoated or coated 25-nm gold nanoparticles, respectively. The TNF:IL12 molar ratio used to prepare the trifunctional nanodrugs is indicated in parentheses. **b** Binding of different nanodrugs (*iso1*Au, Au/TNF, Au/IL12, *iso1*Au/IL12, *iso1*Au/TNF, and *iso1*Au/TNF + IL12) to αvβ3, soluble TNF receptor Type-II (sTNF-R2, Enbrel), and anti-IL12 antibody (mAb C15.6) adsorbed onto nitrocellulose filters, as detected by silver staining of gold nanoparticles. The *iso1*Au/TNF + IL12 used in this experiment was prepared using a TNF:IL12 molar ratio of 4:1. **c** Binding of nanodrugs to microtiter plates coated with or without αvβ3, as detected using a biotinylated antibody anti-IL12 (mAb C17.8) or with anti-TNF antibodies followed by HRP-labeled streptavidin or HRP-labeled secondary antibodies, respectively. Schematic representation of the assays (upper panels). The results of a representative experiment are shown (mean ± SE of duplicates). The TNF:IL12 molar ratio used to prepare the nanodrugs is indicated in parentheses

*Iso1*-coated gold Nps (*iso1*Au) bearing different amounts of TNF and IL12 (called *iso1*Au/TNF + IL12; trifunctional) were prepared by incubating 25-nm Au nanospheres with solutions containing TNF and IL12 in different molar ratios (1:4.5 or 4:1 molar ratio) and fixed amounts of *iso1*-albumin conjugate (*iso1*-HSA) and PEG (see “Methods”). Bifunctional (*iso1*Au/TNF, *iso1*Au/IL12, Au/TNF + IL12) and monofunctional (Au/TNF and Au/IL12) nanodrugs were also prepared by omitting one or two components, respectively, in the incubation mixtures. UV–visible spectrophotometric analysis of the mono-, bi-, and trifunctional products showed similar absorption spectra, as judged from the λ_max_ absorption values, peak width at 75% height (PW75), and 650/530 nm ratio (Fig. 1a, Additional file 1: Figure S1, and Table 1). Furthermore, dynamic light scattering analysis of bifunctional and trifunctional products showed similar average diameters (approximately 43–44 nm) (Table 1), suggesting that these nanodrugs essentially consisted of monodispersed Nps. Accordingly, transmission electron microscopy showed that all nanodrugs were made of gold nanospheres with a maximal diameter of about 28 nm (Additional file 1: Figure S2). Thus, all nanodrugs had very similar physical properties.

**Table 1:** Characterization of mono-, bi-, and trifunctional nanodrugs by UV–visible spectroscopy (UV–Vis) and dynamic light scattering (DLS)

Nanodrug	UV-Vis^b^			DLS^c^
	λ_max_ (nm)	*PW 75%*	A_650_ nm/A_530_ nm	Radius (nm)
Monofunctional				
Au/IL12	528	57	0.071	17.9 ± 2.3
Au/TNF	528 ± 2	58.1 ± 0.1	0.09 ± 0.01	22.1
Bifunctional				
*iso1*Au/IL12	529.3 ± 0.9	54.5 ± 0.7	0.087 ± 0.02	21.9 ± 3.2
*iso1*Au/TNF	528.6 ± 0.3	59.0 ± 0.7	0.095 ± 0.01	21.6 ± 3.9
Au/TNF + IL12^a^ *(4:1)*	531	52	0.111	–
Trifunctional				
*iso1*Au/TNF + IL12^a^ *(1:4.5)*	529.0 ± 1.0	53.9 ± 0.4	0.113 ± 0.02	22.11 ± 2.6
*iso1*Au/TNF + IL12^a^ *(4:1)*	531.9 ± 0.9	55.8 ± 0.3	0.120 ± 0.01	–
Uncoated Au Nps	524.5 ± 0.6	52.0 ± 4.0	0.065 ± 0.02	11.56 ± 1.4

^a^Prepared using the indicated TNF:IL12 molar ratio. See “Methods”

^b^*λmax*: wavelength of peak absorbance; *PW 75%*: peak width at 75% height; A_650 nm_/A_530 nm_: absorbance ratio. Mean ± SD of two different preparations

^c^Mean ± SD of 3 measurements

To assess whether *iso1*, TNF and IL12 were indeed loaded on gold Nps, we measured the capability of all the products to bind αvβ3, sTNF-R2 and anti-IL12 antibodies. We first evaluated the capability of each nanodrug to bind nitrocellulose filters spotted with αvβ3, sTNF-R2 (Enbrel) or anti-IL12 mAb C15.6. Mono-, bi- and trifunctional nanoparticles recognized spots on nitrocellulose with patterns consistent with their expected functional properties (Fig. 1b). Next, we evaluated the capability of each nanodrug to form molecular sandwiches in two integrin-antibody sandwich assays based on (a) αvβ3 and anti-IL12 mAb C17.8 and (b) αvβ3 and polyclonal anti-TNF antibodies. As expected, bi- and trifunctional nanoparticles, but not monofunctional nanoparticles, formed molecular sandwiches with αvβ3 and relevant anti-IL12 or anti-TNF antibodies in the sandwich assays (Fig. 1c), suggesting that the different components of the bi- and trifunctional nanodrugs are accessible to multivalent heterotypic interactions*.*

Overall, these results indicate that the bi- and trifunctional nanodrugs were loaded with *iso1*-HSA, TNF and IL12, as hypothesized, and that *iso1* and TNF preserved their receptor binding properties after coupling to nanogold. In other words, nanogold can be successfully loaded with *iso1*-HSA, TNF and/or IL12.

### Assessment of the biological activity of IL12 and TNF loaded on gold nanoparticles

**Fig. 2:**
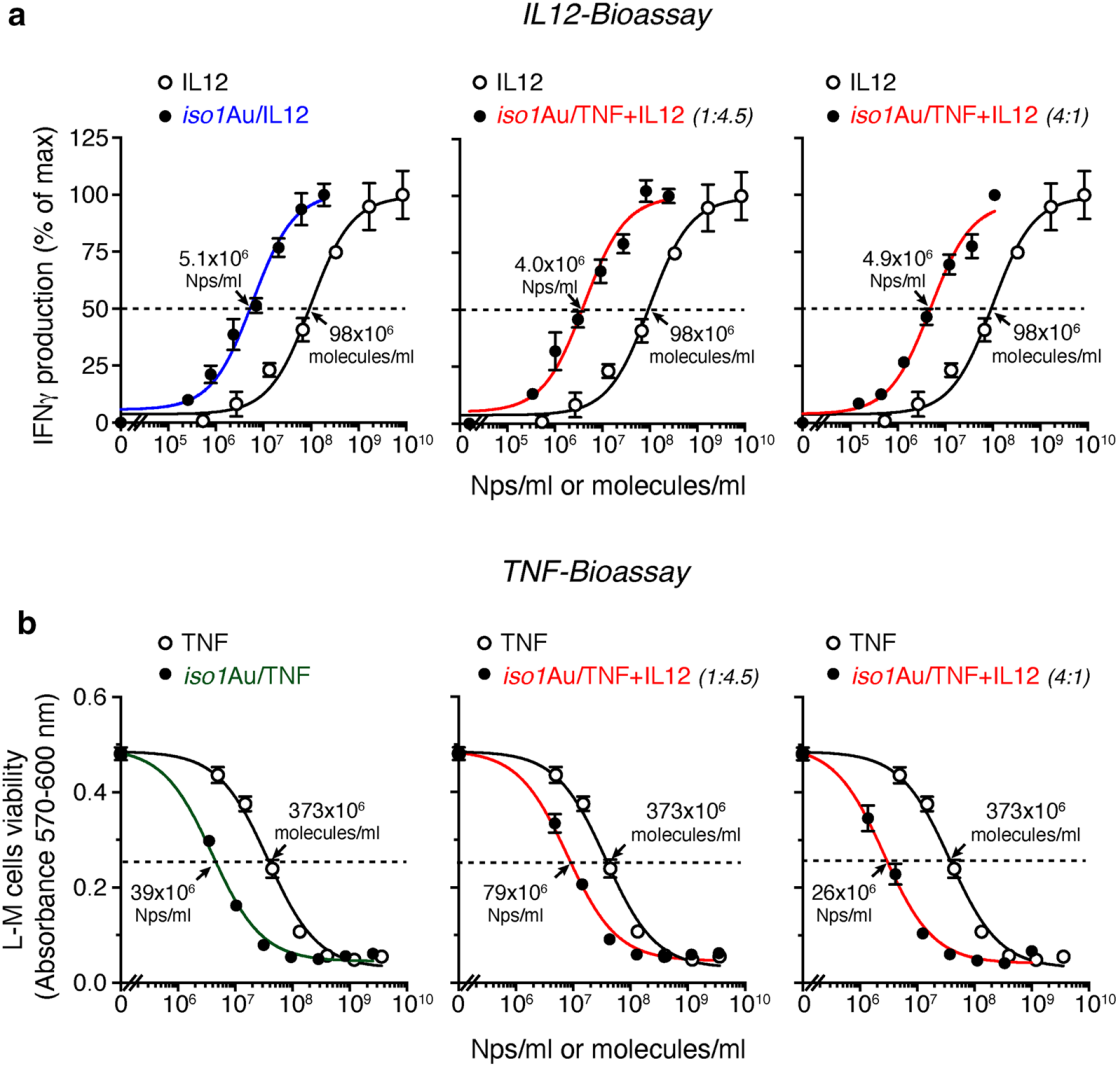
Biological effects of bi- and trifunctional nanodrugs on IFNγ production by splenocytes (**a**) and L–M cell viability (**b**). **a** Effect of nanodrugs and IL12 on IFNγ production by murine splenocytes. Murine splenocytes (200,000 cells in 200 µl of culture medium) were cultured in complete medium supplemented with 125 U/ml of IL-2 and variable amounts of nanodrugs or IL12 for 5 days. IFNγ production in pooled cell supernatants (prepared in quadruplicate) was determined by ELISA (in duplicate). One representative experiment is shown (mean ± SE). The cumulative data of 4–12 experiments are reported in Table 2. The TNF:IL12 ratio used to prepare the trifunctional nanodrug is indicated in parentheses. Arrows, effective concentration 50 (EC_50_). **b** Cytotoxic effects of nanodrugs and TNF on L–M cells. L–M cells (30,000 cells in 100 µl of culture medium) were cultured in complete medium supplemented with 2 µg/ml of actinomycin D and the indicated amounts of nanodrugs or TNF for 20 h at 37 °C and 5% CO_2_. Cell viability was quantified using the PrestoBlue Cell Viability Reagent. One representative experiment is shown (mean ± SE, of triplicates). The cumulative data of 3–7 experiments are reported in Table 2

Next, we quantified the number of IL12 or TNF molecules loaded onto nanogold and assessed their biological activity. Regarding IL12, we measured the capability of *iso1*Au/IL12 and *iso1*Au/TNF + IL12 to bind anti-IL12 antibodies by sandwich IL12-ELISA (immunoassay) and to promote IFNγ release from cultured murine splenocytes (bioassay). Both assays were calibrated with IL12. The immunoreactivity of each bi- or trifunctional nanoparticle was equivalent to that of 5–19 molecules of IL12, depending on the preparation protocol (Table 2). In particular, *iso1*Au/IL12 showed 6.4 molecules of IL12/Np, whereas *iso1*Au/TNF + IL12 prepared with 4:1 and 1:4.5 molar ratios of TNF/IL12 showed 4.7 and 19.1 molecules/Np of IL12, respectively. Interestingly, when these drugs were tested using the IFNγ-release bioassay, their potency was equivalent to 47, 35 and 40 molecules/Np of IL12, respectively (Table 2). The apparent discrepancy between the immunoassay and bioassay may be due to a more efficient engagement of IL12 receptors by gold-bound IL12 compared with “free” IL12, owing to multivalent interactions of each nanoparticle with individual cells. Notably, the EC_50_ of the two *iso1*Au/TNF + IL12 preparations (4:1 and 1:4.5 molar ratios) in the IL12 bioassay corresponded to 4.8 × 10^6^ and 4.2–4.8 × 10^6^ Nps/ml, respectively, despite the different number of immunoreactive IL12 molecules/Np (Fig. 2 and Table 2). Considering that 200,000 cells/200 µl were seeded in each microtiter plate well, these EC_50_ values correspond to 4–5 Nps/cell. These data suggest that the binding of a few nanoparticles/splenocyte is sufficient to trigger IFNγ release.

**Table 2:** Quantification of TNF, IL12 and PEG molecules loaded on gold nanoparticles

Nanodrug^a^	IL12			TNF		PEG
	IL12-ELISA	IL12-Bioassay^d^		TNF-Bioassay^e^		PEG-ELISA
	IL12 (molecules/Np)	IL12 (molecules/Np)	EC_50_ (Nps/ml)	TNF (molecules/Np)	EC_50_ (Nps/ml)	PEG (molecules/Np)
Bifunctional						
*iso1*Au/IL12	6.4 ± 1.6 (*n* = *4*)^c^	47 ± 10 (*n* = *4*)	3.6 (± 0.8) × 10^6^ (*n* = *4*)	–	-	~ *4* (*n* = *1*)
*iso1*Au/TNF	–	–	–	6.8 ± 1.0 (*n* = *3*)	52 (± 8) × 10^6^ (*n* = *3*)	~ *3* (*n* = *1*)
Trifunctional						
*iso1*Au/TNF + IL12 (1:4.5)	19.1 ± 3.8 (*n* = *5*)	35 ± 9 (*n* = *4*)	4.8 (± 1.2) × 10^6^ (*n* = *4*)	2.4 ± 0.3 (n = 7)	146 (± 22) × 10^6^ (*n* = *7*)	~ *7* (*n* = *1*)
*iso1*Au/TNF + IL12 (4:1)	4.7 ± 0.6 (*n* = *8*)	40 ± 7 (*n* = *4*)	4.2 (± 0.7) × 10^6^ (*n* = *4*)	14.1 ± 5.1 (*n* = *4*)	25 (± 9) × 10^6^ (*n* = *4*)	–

Protein^b^			EC_50_ (molecules/ml)		EC_50_ (molecules/ml)	
IL12		–	169 (± 40) × 10^6^ (*n* = *12*)	–	–	–
TNF	–	–	–	–	352 (± 9) × 10^6^ *(n* = *7*)	–

The italic numbers indicate the number of independent experiments

Mean ± SE

^a^Prepared as described in “Methods” section. The TNF:IL12 molar ratio used for *iso1*Au/TNF + IL12 preparation is indicated in parentheses

^b^Recombinant murine IL12 and TNF

^c^*n*, number of independent experiments

^d^IL12-induced secretion of IFNγ by murine splenocytes (reference standard, recombinant IL12) (see “Methods”). EC_50_: effective concentration 50

^e^L–M cell cytotoxicity assay (reference standard, recombinant TNF) [29]

To quantify the amount of bioactive TNF loaded onto *iso1*Au/TNF and *iso1*Au/TNF + IL12, we tested their cytotoxic effects against murine L–M fibroblasts using TNF as a reference standard. The potency of each *iso1*Au/TNF Np was equivalent to that of 6.8 TNF molecules, whereas that of *iso1*Au/TNF + IL12 Nps prepared with 4:1 and 1:4.5 molar ratios of TNF/IL12 was equivalent to 14.1 and 2.4 TNF molecules, respectively (Fig. 2b and Table 2).

Overall, these results establish the feasibility of the multifunctional cytokine nanoformulation based on gold Nps.

### *Iso1*Au/TNF,* iso1*Au/IL12 and *iso1*Au/TNF + IL12 exert anti-tumor effects in a murine model of fibrosarcoma

**Fig. 3:**
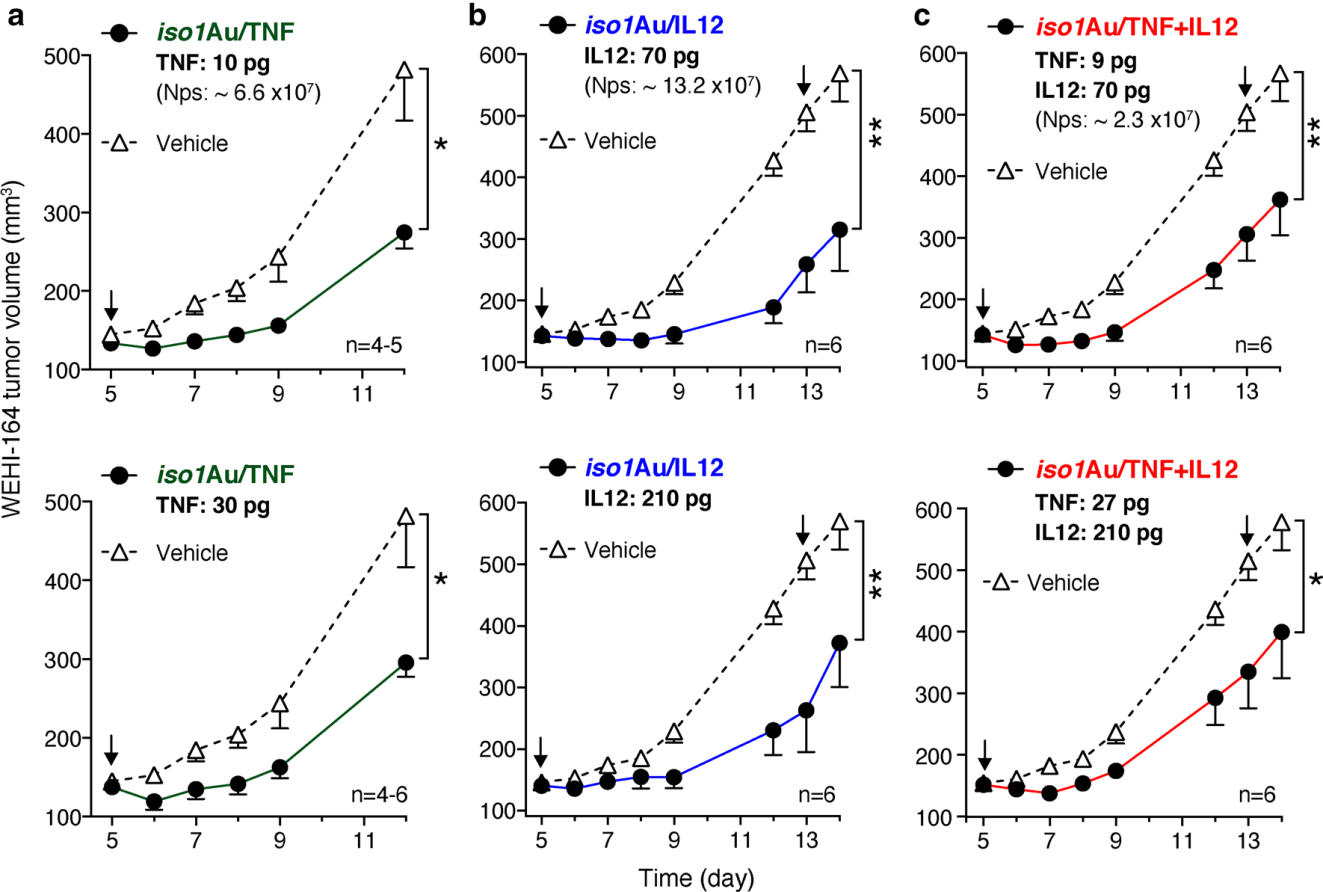
Anti-tumor effects of bi- and trifunctional nanodrugs in the WEHI-164 fibrosarcoma model. Tumor-bearing mice were treated at the indicated times (arrows) after tumor implantation with the indicated doses of *iso1*Au/TNF (**a**), *iso1*Au/IL12 (**b**) or *iso1*Au/TNF + IL12 (**c**) (TNF:IL12 molar ratio, 1:4.5) (i.v.). The indicated doses correspond to immunoreactive IL12, as detected by ELISA, or bioactive TNF, as determined by the L–M cytolytic assay. The tumor volumes are shown (mean ± SE, 4–6 mice per group). *P < 0.05; **P < 0.01 by Mann–Whitney analysis of the area under the curve for each tumor with GraphPad Prism software

We then analyzed the anti-tumor activity of bi- and trifunctional nanodrugs using immunocompetent mice bearing subcutaneous WEHI-164 fibrosarcomas. In previous studies, we found that *iso1*Au/TNF and *iso1*Au/IL12 exerted maximal anti-tumor activity when injected at doses corresponding to approximately 5–15 pg of TNF [20, 21] and 20–70 pg of IL12 [23], respectively. Thus, for in vivo studies, we used *iso1*Au/TNF + IL12 prepared using a TNF:IL12 molar ratio of 1:4.5, i.e., with less TNF than IL12 (2.4 and 19.1 molecules/Np, respectively). The administration of doses of *iso1*Au/TNF, *iso1*Au/IL12, or *iso1*Au/TNF + IL12 equivalent to approximately 10 pg of TNF or 70 pg of IL12 induced similar anti-tumor effects (Fig. 3, upper panels). Similar anti-tumor effects were also observed using three-fold higher doses (Fig. 3, lower panels). Notably, in previous studies, we have shown that comparable doses of TNF and IL12 were completely inactive in the same model [20, 21, 23]. These data indicate that the *iso1*Au nanoformulation of these cytokines increases their anticancer activity. Although no greater effects were observed with the trifunctional Nps in the WEHI-164 fibrosarcoma model, compared to bifunctional Nps, these data indicate that this nanodrug is biologically active in vivo.

### *Iso1*Au/TNF and *iso1*Au/TNF + IL12, but not iso1Au/IL12, exert synergistic effects with doxorubicin in a murine model of fibrosarcoma

**Fig. 4:**
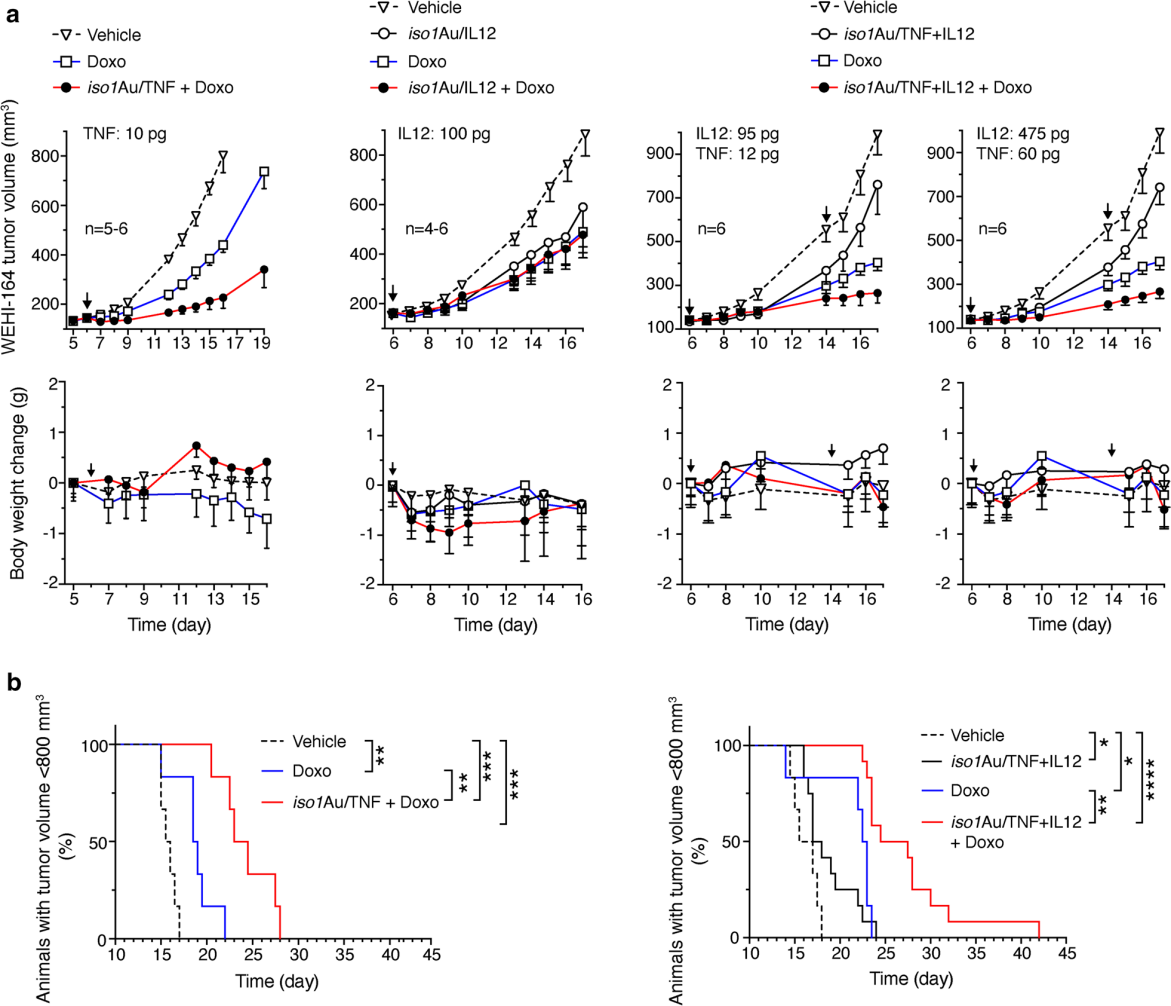
Effect of bi- and trifunctional nanodrugs alone or in combination with doxorubicin (Doxo). WEHI-164 tumor-bearing mice were treated (i.v.) at the indicated times after tumor implantation (arrows) with the indicated doses of *iso1*Au/TNF, *iso1*Au/IL12, *iso1*Au/TNF + IL12 and Doxo (150 µg). The indicated doses correspond to immunoreactive IL12, as detected by ELISA, or bioactive TNF, as determined by the L–M cytolytic assay. **a** Tumor volume (upper panels) and body weight changes (lower panels) (mean ± SE, 4–6 mice per group). **b** Kaplan–Meier curves of Doxo alone and in combination with *iso1*Au/TNF or *iso1*Au/TNF + IL12. The cumulative data of the two experiments performed with *iso1*Au/TNF + IL12 (**a** right panels) are reported. *P < 0.05; **P < 0.01, ***P < 0.001, ****P < 0.0001 by log-rank (Mantel-Cox) test

The capability of bifunctional and trifunctional nanodrugs to synergize with doxorubicin (Doxo) was investigated using the same tumor model. The administration of 150 µg of Doxo alone induced a significant delay in tumor growth (Fig. 4a upper panels, and Fig. 4b). This effect was significantly increased when combined with *iso1*Au/TNF or *iso1*Au/TNF + IL12 but not with *iso1*Au/IL12 (Fig. 4a upper panels, and Fig. 4b). None of the tested nanodrugs worsened Doxo-related toxicity, as judged by the animal weight loss (Fig. 4a, lower panels). These results indicate that both bi- and trifunctional Nps bearing TNF can increase the therapeutic index of Doxo.

### *Iso1*Au/TNF promotes doxorubicin penetration in tumor tissues and causes vascular damage at later time points

**Fig. 5:**
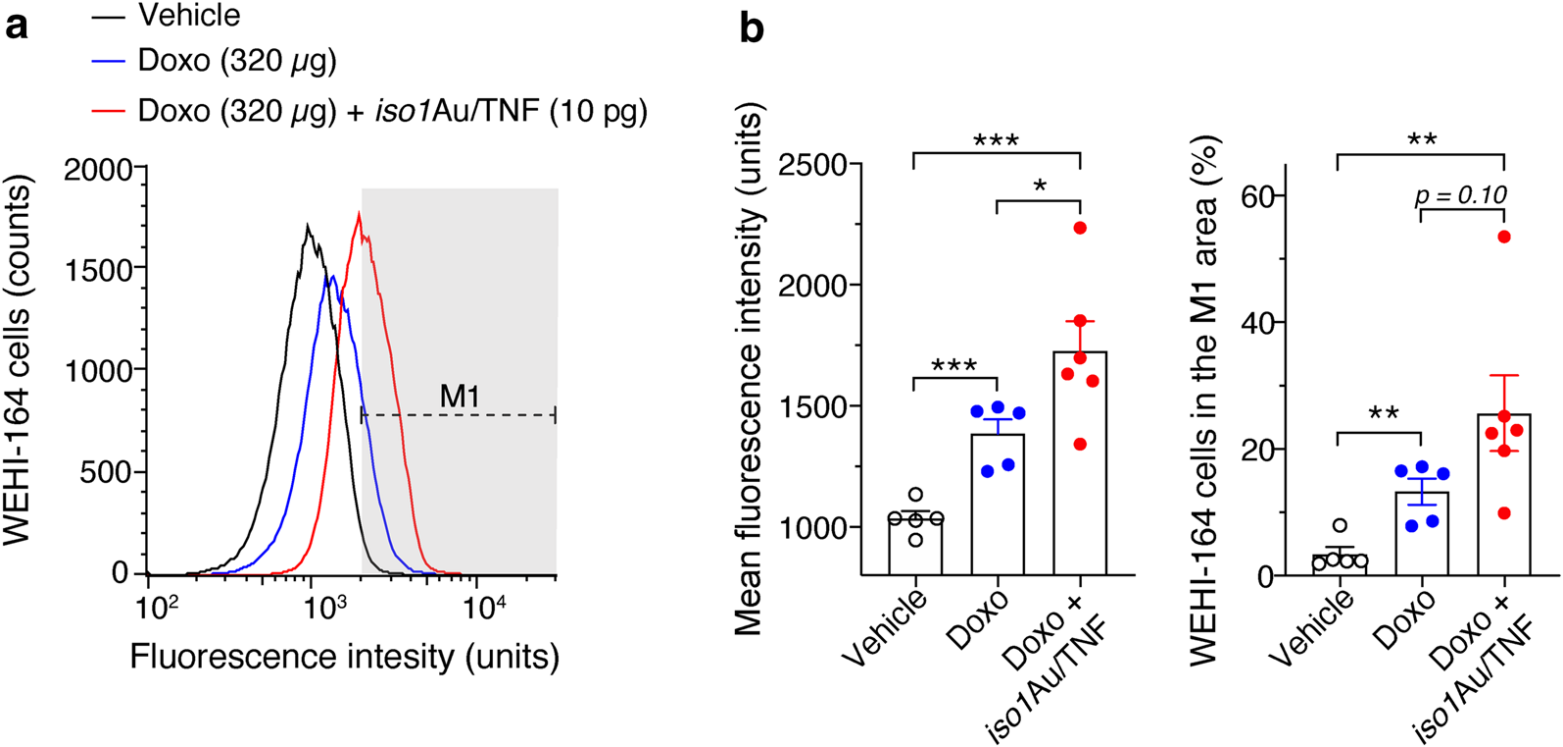
*iso1*Au/TNF increases the tumor cell uptake of Doxo. WEHI-164 tumor-bearing mice (approximately 0.4–0.8 cm in tumor diameter) were treated i.v. with vehicle or the indicated dose of doxo alone or in combination with *iso1*Au/TNF. After 2 h, tumors were recovered, weighed, minced, and processed as described previously [3] to obtain a single-cell suspension, and analyzed by flow cytometry. **a** Representative flow cytometry analysis of WEHI-164 tumor cells recovered from mice treated as indicated. The *M1* dotted line indicates the fluorescence interval considered positive. **b** Mean fluorescence intensity (left) and percentage of positive cells in the M1 area (right). Cumulative results of 2 independent experiments (mean ± SE, 2–3 mice/experiment). *P < 0.05; **P < 0.01, ***P < 0.001, by two-tailed t-test

**Fig. 6:**
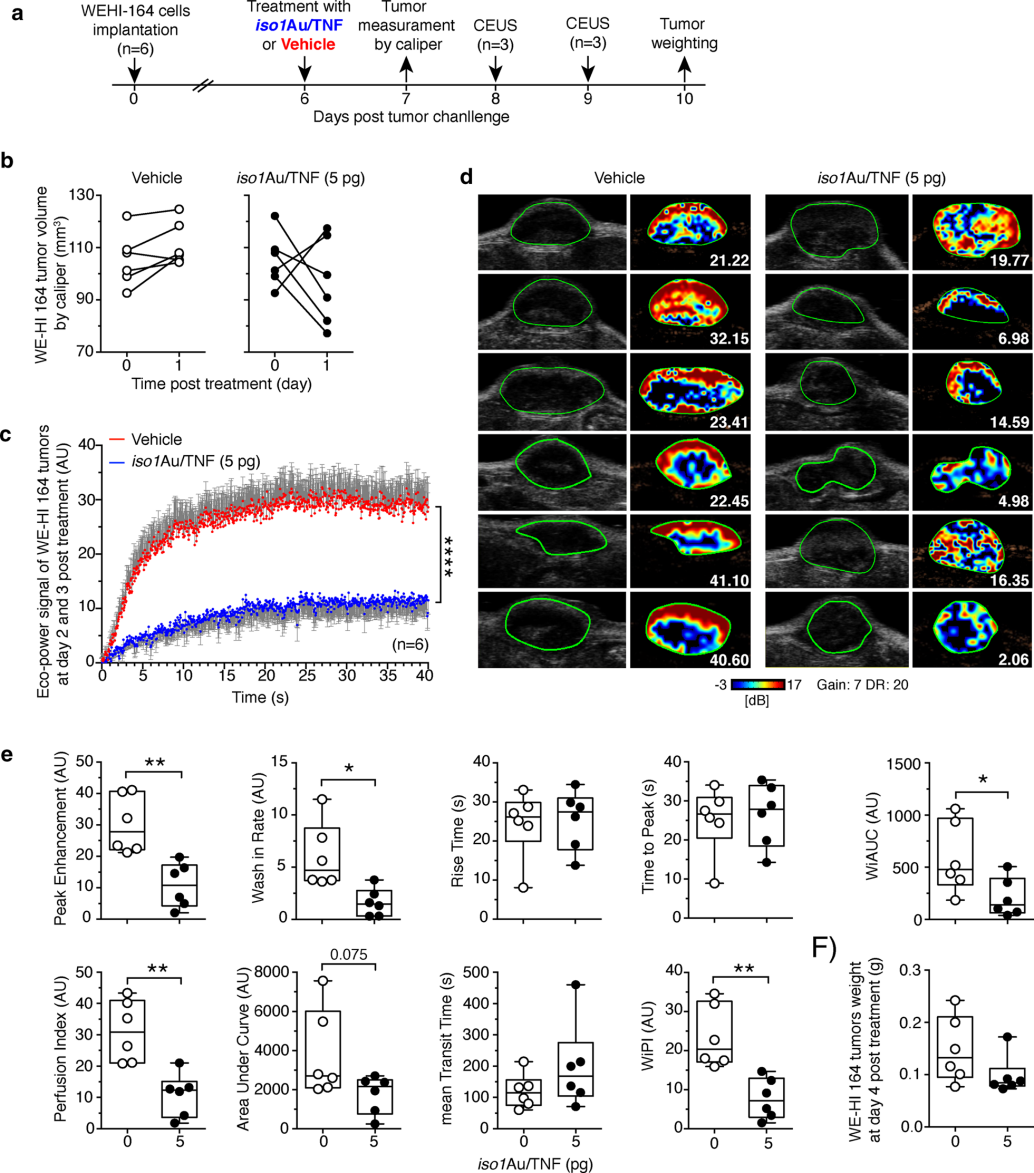
*Iso1*Au/TNF reduces tumor perfusion. WEHI 164 tumor-bearing mice (n = 6/group) were treated i.v. with the indicated dose of *iso1*Au/TNF at day 6 after tumor implantation and were analyzed by contrast-enhanced ultrasound (CEUS) at day 8 (3 mice) and day 9 (3 mice). MicroMarker Contrast Agent was injected at time 0, and its uptake was recorded for 40 s. **a** Scheme of the experiment. **b** Effect of *iso1*Au/TNF (5 pg) or vehicle on tumor growth before and after treatment (day 1), as determined by calipers. **c** Time-intensity curves (mean ± SE); ****p < 0.0001, by two-way ANOVA. **d** Grayscale tumor images (left) and color-coded peak enhancement (PE) maps of representative mice. Red and blue areas correspond to high and low perfused tumor areas, respectively. Numbers, PE values. **e** Quantitative analysis of tumor perfusion with microbubbles performed using VevoCQTM software. CEUS parameters were calculated using a region of interest (ROI, green lines in **d**) corresponding to the entire tumor area. Box plots of CEUS parameters with 5–95 percentiles and medians are shown; *P < 0.05; **P < 0.001 by *two-tailed t-test*. Images were taken at regions with a maximum tumor diameter. **f** Tumor weight at day 10

The mechanism underlying the anti-tumor activity of *iso1*Au/TNF and its synergism with Doxo was then investigated. Considering that TNF can increase tumor vascular permeability and reduce drug penetration barriers, we tested the hypothesis that this nanodrug increased Doxo penetration in tumors. To this aim, we exploited the fact that Doxo is a fluorescent compound and that the fluorescence intensity of the tumor cells recovered from animals after treatment indicates the amount of Doxo that penetrated tumors [3]. As expected, *iso1*Au/TNF increased the fluorescence intensity and the percentage of positive cells recovered from tumors 2 h after treatment (Fig. 5). Considering that TNF can also have vascular-damaging effects [31, 32], we then analyzed the impact of *iso1*Au/TNF on tumor perfusion using contrast-enhanced ultrasound analysis with microbubbles (CEUS). A marked reduction in the signal/time area under the curve (AUC) and peak enhancement of microbubbles was observed 2–3 days after treatment (Fig. 6). This and the above findings suggest that *iso1*Au/TNF reduces drug penetration barriers in tumors at early time points, favoring drug extravasation, and causes vascular shutdown at later time points in some tumor areas. Both mechanisms, together with the anti-cancer effects of Doxo, may have contributed to the overall anti-tumor activity of the combined therapy.

## Discussion

This work shows that gold nanospheres coated with the *iso1* peptide can be used as a versatile platform for single- or multicytokine delivery to the tumor vasculature. In particular, the results of in vitro studies show that bi- or trifunctional nanodrugs consisting of nanogold coated with *iso1*, bioactive TNF and/or IL12 can be easily prepared by incubating 25-nm gold nanospheres with *iso1*-HSA, TNF and IL12 in different proportions. Notably, the results of in vitro bioassays show that the “nanoformulated” cytokines (IL12 and TNF) trigger biological effects more efficiently than the corresponding “free” cytokines. For example, 35- to 47-fold lower amounts of nanoformulated IL12, compared to IL12, were sufficient to evoke the secretion of IFNγ from murine splenocytes. This phenomenon was not related to *iso1*-mediated targeting mechanisms of nanoformulated cytokines, because *iso1*Au/IL12 and Au/IL12 showed similar potencies in this assay (*data not shown*). More likely, this phenomenon was related to high-avidity interactions engaged by multivalent nanoparticles with IL12 membrane receptors. Accordingly, enhanced effects have also been observed with TNF upon nanogold formulation.

The enhanced cytokine activity, together with peptide- and gold-mediated active and passive targeting mechanisms (which may occur in vivo), may contribute to reducing the dose of cytokines necessary for inducing pharmacological effects. According to this view, we found that extremely low, nontoxic doses of *iso1*Au/TNF or *iso1*Au/TNF + IL12, corresponding to 3–6 × 10^7^ nanoparticles (equivalent to 5–10 pg of TNF), were sufficient to exert synergistic effects with doxorubicin in the WEHI-164 fibrosarcoma model. If we assume that approximately 1% of the injected dose accumulated in tumors, as typically observed with nanoparticles [33], we estimate that only 3–6 × 10^5^ nanoparticles reached their target in tumors. Considering that 0.1- to 0.2-cm^3^ tumors may contain 1–2 × 10^7^ cells [34], this number of nanoparticles is considerably lower than that of the neoplastic cells present in the tumors at the time of treatment. Endothelial cells, which represent a small fraction of the tumor mass (2–11%) [21, 35] and express the αvβ3 receptor of *iso1* [36, 37], are likely the target of our nanodrugs. Thus, we estimate, from these figures, that the delivery of only 1–4 Nps/endothelial cells is sufficient to induce pharmacological effects. Analysis of doxorubicin uptake by tumor cells, performed 2 h after treatment with *iso1*Au/TNF, showed that this nanodrug enhanced the amount of doxorubicin that reached cancer cells, likely by increasing endothelial permeability and reducing drug penetration barriers. However, analysis of tumor perfusion by contrast-enhanced ultrasound 2–3 days after treatment showed that *iso1*Au/TNF, despite its low dose, could also cause vascular shutdown in some tumor areas. Thus, while *iso1*Au/TNF can reduce doxorubicin penetration barriers at early time points, at later times this nanodrug may also further entrap doxorubicin in tumor tissues by impairing tumor perfusion. The combination of these time-dependent effects on the tumor vasculature may have contributed to the synergism observed with chemotherapy, resulting in a prolonged survival of mice.

In previous studies, we have shown that low doses of NGR-TNF, a peptide-TNF fusion protein that targets the tumor vasculature, are sufficient to cause the disassembly VE-cadherin dependent-adherence junctions and gap formation in the tumor endothelium, thereby increasing vascular permeability [38]. This compound can also enhance the expression of leukocyte adhesion molecules on tumor endothelial cells and induce cytokine/chemokine secretion, thereby enhancing lymphocyte infiltration [39]. TNF can also induce endothelial cell apoptosis, vascular damage, intravascular coagulation and occlusion [40], leading to ischemic and hemorrhagic necrosis [41]. These mechanisms are likely triggered also by *iso1*Au/TNF, as suggested by the increased doxorubicin uptake, 2 h after treatment, and by the reduction of tumor perfusion at later time points.

Other investigators have shown previously that 27-nm nanogold functionalized with TNF (called CYT-6091) can suppress tumor perfusion and increase the antitumor effects of chemotherapy in murine models of mammary carcinoma and in genetically engineered mice with pancreatic ductal adenocarcinoma, when administered at doses equivalent to 3–5 µg of bioactive TNF [42, 43]. Notably, our results show that markedly lower doses of iso1Au/TNF (equivalent to 5–10 pg of bioactive TNF) are sufficient to affect vascular permeability, tumor perfusion and antitumor efficacy of chemotherapy. The extremely low dose of iso1Au/TNF necessary to induce anti-tumor effects may represent an important advantage in terms of toxicity.

*Iso1*Au/TNF and *iso1*Au/TNF + IL12 showed similar anti-tumor effects in the murine fibrosarcoma model, when used alone or in combination with chemotherapy, suggesting that the addition of IL12 does not increase the anti-tumor effects of *iso1*Au/TNF in this model. The rationale for combining TNF and IL12 in a single nanodrug (*iso1*Au/TNF + IL12) relies on the notion that IL12 can induce the release of IFNγ from lymphoid cells, such as NK cells and T cells, a cytokine crucial for the activity of low-dose targeted TNF [26]. Furthermore, in previous studies, we showed that low-dose *iso1*Au/IL12 can significantly increase tumor infiltration by innate immune cells, such as NK and iNKT cells, monocytes, and neutrophils [23]. However, *iso1*Au/IL12 can also induce the release of sTNF-R2 in the tumor microenvironment, an inhibitor of TNF [23]. We speculate that this mechanism may have counterbalanced, to some extent, the potential synergism of these cytokines when combined in a single drug, such as in the case of *iso1*Au/TNF + IL12.

In any case, given that the addition of IL12 to *iso1*Au/TNF did not impair its synergism with chemotherapy in the WEHI fibrosarcoma model and considering our previous studies showing that *iso1*Au/IL12 and TNF can individually support the therapeutic efficacy of adoptive T-cell therapy in other tumor models [23, 44], the trifunctional *iso1*Au/TNF + IL12 might exert synergistic effects with other chemo-immunotherapeutic combinations in different tumors, which is an approach that merits future investigation.

## Conclusion

We have shown that *iso*DGR-coated gold nanospheres can be exploited as a versatile platform for single- or multicytokine delivery to cells of the tumor vasculature and that extremely low doses of *iso*DGR-coated nanoparticles functionalized with TNF or with TNF + IL12 can induce synergistic effects with doxorubicin. Furthermore, the present study establishes the conceptual feasibility of multifunctional cytokine nanoformulations using *iso1*-coated gold nanoparticles, a technology potentially applicable to other cytokines.

## Supplementary Information

**Additional file 1: Scheme S1.** Schematic representation of the protocol used for preparation of *iso1*Au/TNF (A) *iso1*Au/IL12 (B) and *iso1*Au/TNF + IL12 (C). **Figure S1.** UV–Vis absorption spectra of Au/IL12, Au/TNF and Au/TNF + IL12. **Figure S2.** Characterization of *iso1*Au/TNF, *iso1*Au/IL12 and *iso1*Au/TNF + IL12 by transmission electron microscopy (TEM).
