# Supplementary material for: Enhancement of doxorubicin anti-cancer activity by vascular targeting using IsoDGR/cytokine-coated nanogold
Source: J Nanobiotechnology. 2021 May 5;19:128. doi: 10.1186/s12951-021-00871-y (PMC8097910; doi:10.1186/s12951-021-00871-y)
Supplement: Supplementary file 1 — Additional file 1: Scheme S1. Schematic representation of the protocol used for preparation of iso1Au/TNF (A) iso1Au/IL12 (B) and iso1Au/TNF + IL12 (C). Figure S1. UV–Vis absorption spectra of Au/IL12, Au/TNF and Au/TNF + IL12. Figure S2. Characterization of iso1Au/TNF, iso1Au/IL12 and iso1Au/TNF + IL12 by transmission electron microscopy (TEM). [file 12951_2021_871_MOESM1_ESM.docx]

**Additional file 1**

**Enhancement of Doxorubicin Anti-Cancer Activity by Vascular Targeting using IsoDGR/cytokine-coated Nanogold**

Angelo Corti, Angelina Sacchi, Anna Maria Gasparri, Matteo Monieri, Giulia Anderluzzi, Barbara Colombo, Alessandro Gori, Anna Mondino and Flavio Curnis

**Additional SCHEMES and FIGURES**

- **Scheme 1**
- **Figure S1**
- **Figure S2**


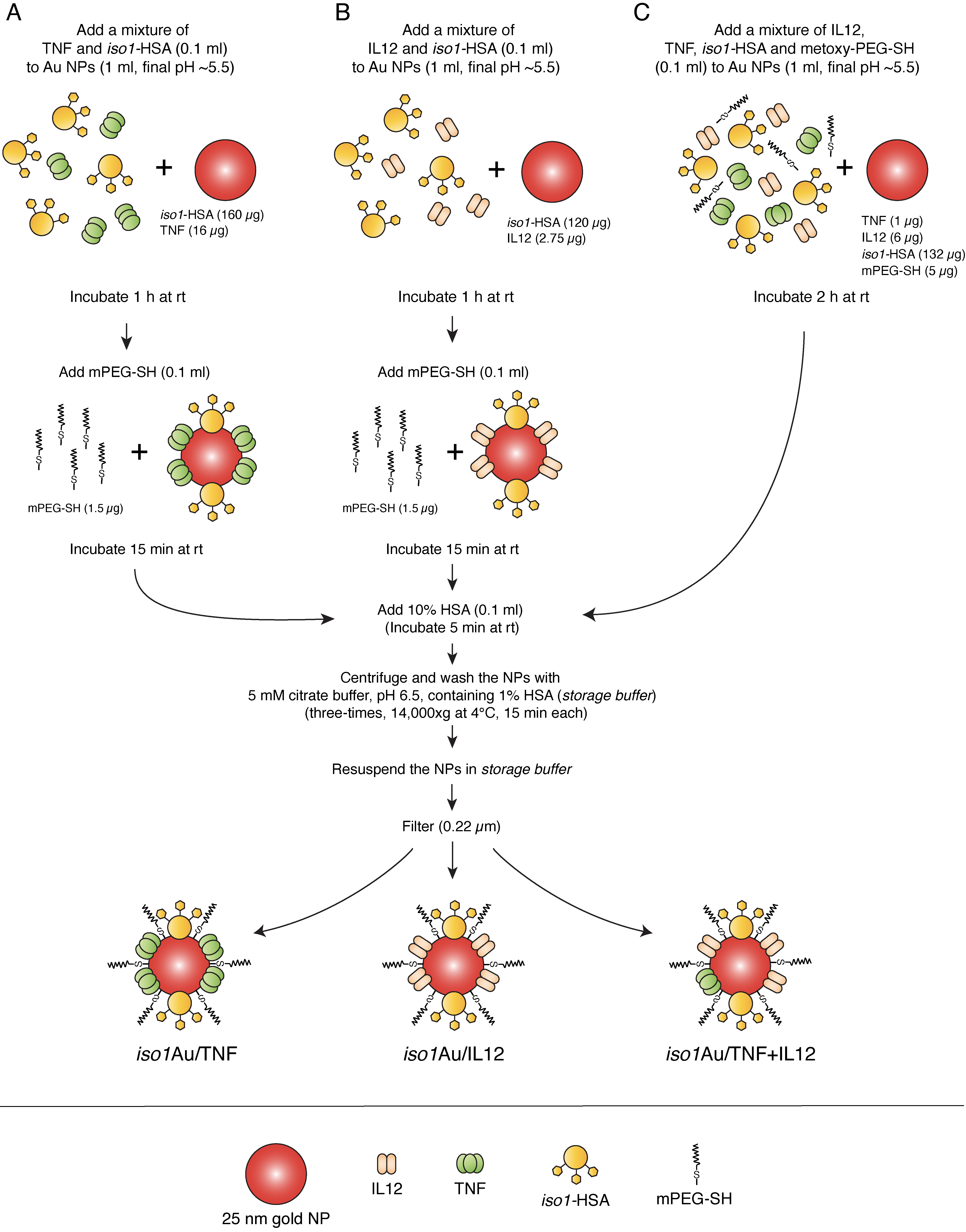


**Scheme S1. Schematic representation of the protocol used for preparation of *iso1*Au/TNF (A) *iso1*Au/IL12 (B) and *iso1*Au/TNF+IL12 (C).**

Control nanoparticles (Au/TNF, Au/IL12 and Au/TNF+IL12) were also prepared according to the indicated scheme using SMCC-activated HSA and quenched with βmercaptoethanol instead of *iso1*-HSA.


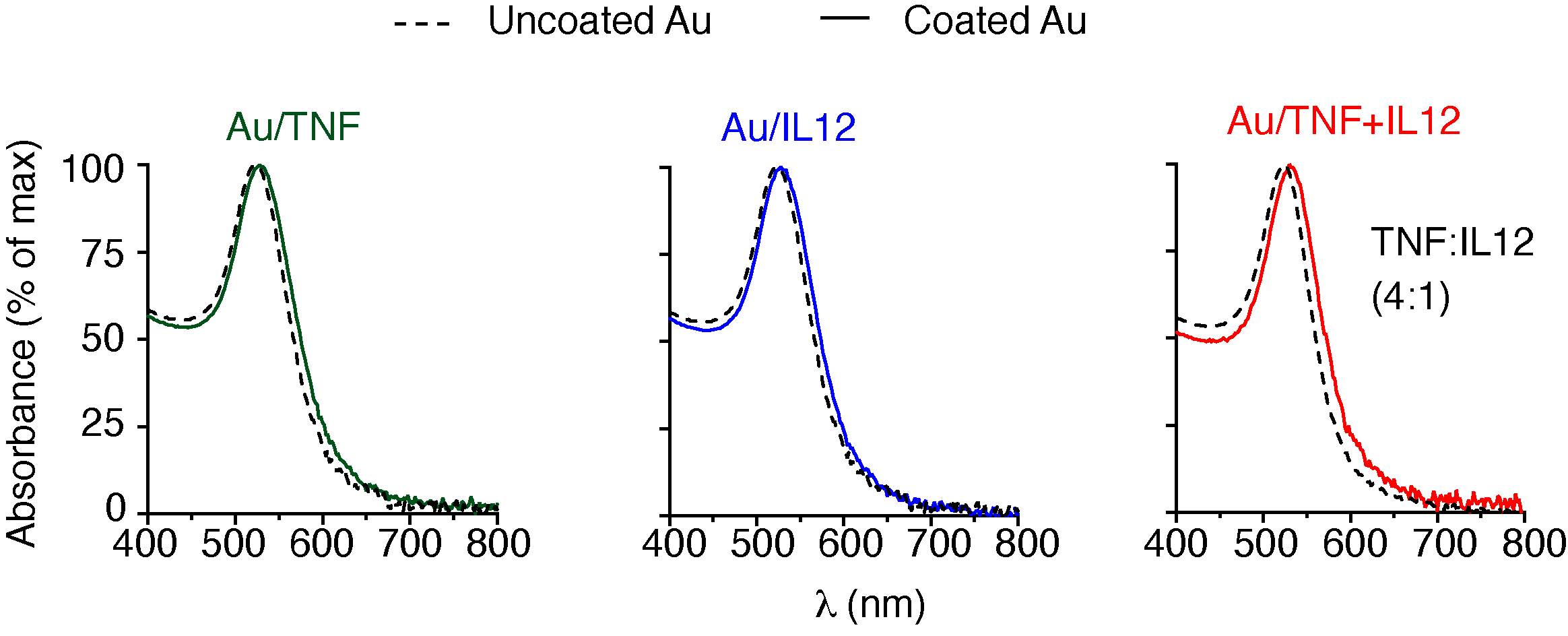


**Figure S1. UV–Vis absorption spectra of Au/IL12, Au/TNF and Au/TNF+IL12.**

Dotted line corresponds to uncoated 25 nm gold nanoparticles. The TNF/IL12 molar ratio used for the preparation of Au/TNF+IL12 is indicated (4:1).


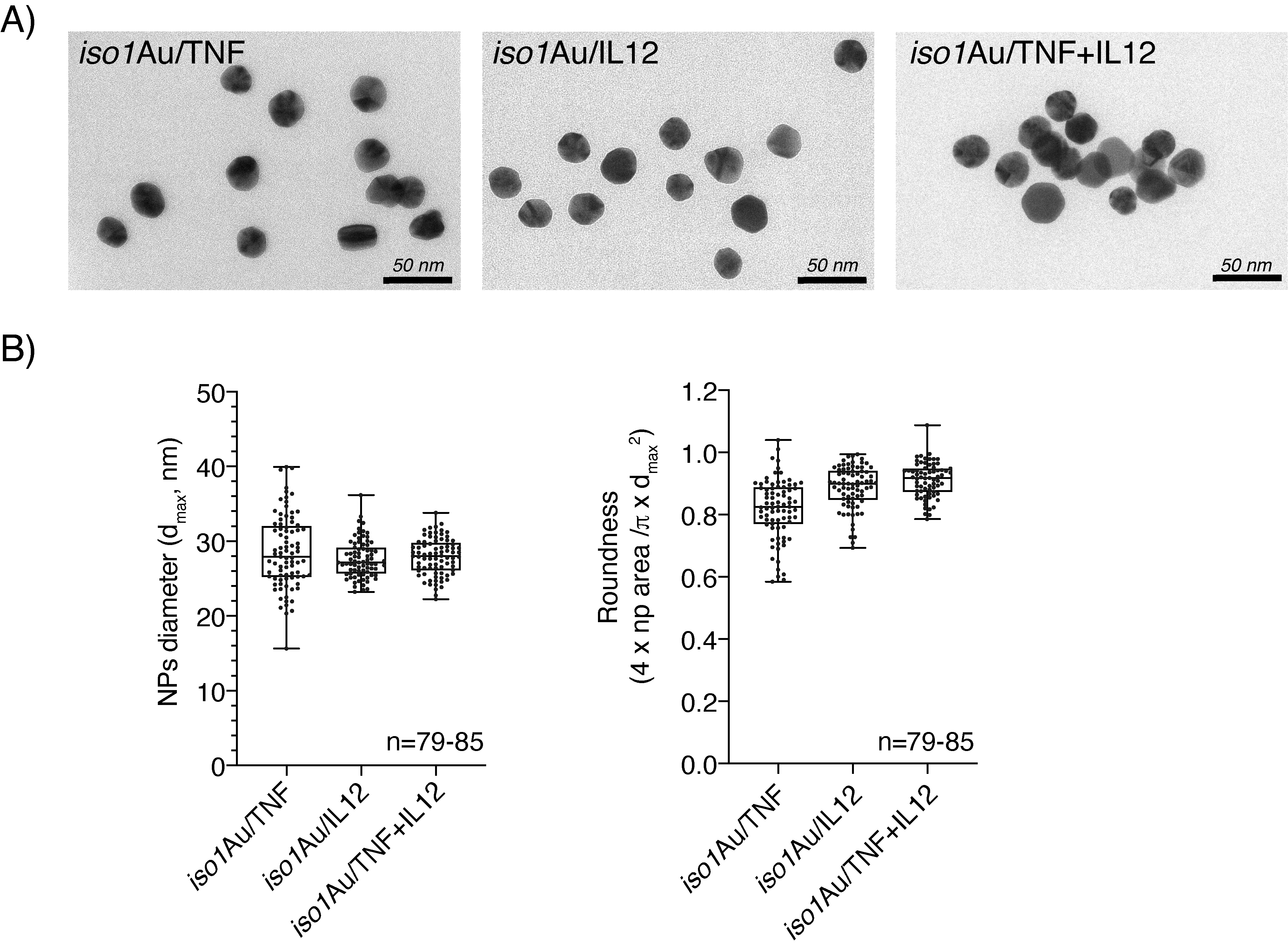


**Figure S2. Characterization of *iso1*Au/TNF, *iso1*Au/IL12 and *iso1*Au/TNF+IL12 by transmission electron microscopy (TEM).**

**A**) Representative microphotographs of each nanodrug and (**B**) morphometric quantification of nanoparticle maximal diameter (*Feret's Diameter*) and shape (*roundness*). Morphometric analysis of the indicated number of nanoparticles shows that *iso1*Au/TNF, *iso1*Au/IL12 and *iso1*Au/TNF+IL12 consist of gold nanospheres with maximal diameters of 28.5±4.8 nm, 27.9±2.5 nm and 27.5±2.3 (mean±SD), respectively, and with a roundness value of 0.82±0.09, 0.89±0.07 and 0.91±0.06, respectively (a roundness value of 1 correspond to a perfect circle). Box plots with medians, interquartiles and 5-95 percentiles are shown.
